# Diffuse Large B Cell Lymphoma Mimicking Granulomatosis with Polyangiitis

**DOI:** 10.1155/2016/1041787

**Published:** 2016-05-18

**Authors:** Mohammad E. Naffaa, Alexander P. Rozin, Netanel Horowitz, Ofer Ben-Itzhak, Yolanda Braun-Moscovici, Alexandra Balbir-Gurman

**Affiliations:** ^1^The B. Shine Rheumatology Unit, Rambam Health Care Campus, 31096 Haifa, Israel; ^2^Rappaport Faculty of Medicine, Technion-Israel Institute of Technology, 31096 Haifa, Israel; ^3^Hematology Institute, Rambam Health Care Campus, 31096 Haifa, Israel; ^4^Pathology Institute, Rambam Health Care Campus, 31096 Haifa, Israel

## Abstract

In a patient with systemic multiorgan disease with overlapping features, the differential diagnosis included infectious diseases, malignancies, and systemic autoimmune or inflammatory diseases. We present an unusual case of a young male with B cell lymphoma who presented with symptoms mimicking systemic vasculitis and review the existing literature.

## 1. Introduction

Granulomatosis with polyangiitis (GPA) is a small and medium-sized vessels vasculitis which is highly associated with positive anti-neutrophil cytoplasmic antibodies (ANCA), mainly with antibodies against proteinase 3 (PR3) [1]. Among reported clinical manifestations of GPA, necrotizing granulomatous inflammation in the upper respiratory tract, pulmonary nodules (often with cavitations), and necrotizing glomerulonephritis are prominent; their combination is a classic GPA triad [1]. Involvement of other organs and systems, such as the skin, eyes, ears, and peripheral nervous system, is less specific but definitely is not rare. The involvement of gastrointestinal tract (GIT) in GPA is infrequent [2].

## 2. Case Presentation

**Figure 1:**
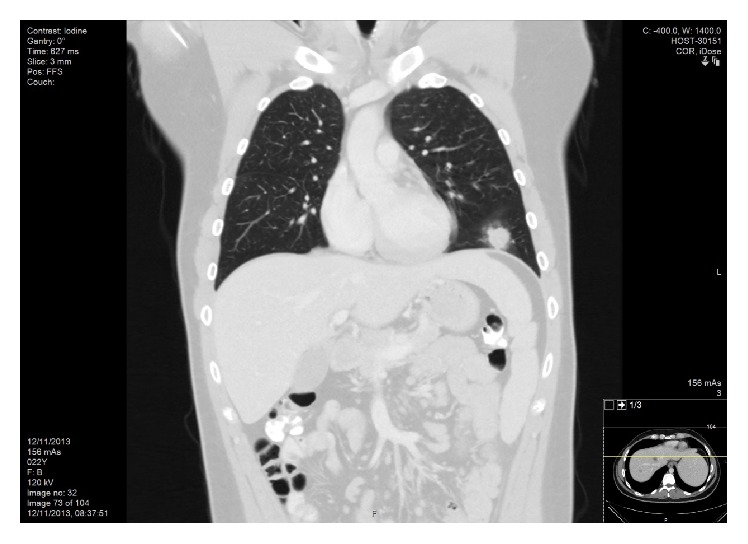
Coronal reconstruction of thoracic CT scan in lung window irregular pulmonary nodule with surrounding ground glass opacity in the left lower lobe of the lung.

A 22-year-old male was admitted because of fever (39.5°C), weakness, excessive sweating, vomiting, and weight loss during the previous month. His medical history was unremarkable, and physical examination did not show any pathological signs. Laboratory data revealed white blood count 2100 (WBC, 4000–10800/*μ*L), hemoglobin 12.5 (Hb, 13–17 g/dL), and platelet count 113000 (PLT, 130000–350000/*μ*L); aspartate aminotransferase 65 (AST, 5–40 U/L), alanine aminotransferase 63 (ALT, 30–65 U/L), alkaline phosphatase 123 (AlkPh, 30–115 U/L), lactate dehydrogenase 997 (LDH, 60–225 U/L), creatinine 0.73 (0.4–1.3 mg/dL), blood urea nitrogen 8 (BUN, 5–20 mg/dL), creatine kinase 20 (CK, 35–300 U/L), albumin 3.9 (3.4–5 g/dL), and ferritin 469 (15–300 ng/mL); erythrocyte sedimentation rate 24 mm/1 hour (ESR, <20 mm/1 hour) and C-reactive protein 18 mg/L (CRP, 0–5 mg/L). Coagulation profile and thyroid function tests were normal. Urine dip-stick was negative for protein or blood. Blood, urine, throat, and stool cultures were all sterile. Serology tests, including human immunodeficiency virus, hepatitis B and C virus,* Cytomegalovirus*, Epstein Barr virus (EBV),* Rickettsia*,* Chlamydia*,* Toxoplasma*,* Brucella*, and Q-fever, were negative. Anti-nuclear (ANA) and anti-dsDNA antibodies were positive; anti-cardiolipin, and B_2_-glycoprotein antibodies and cryoglobulins were negative; complements C3 and C4 levels were normal; protein electrophoresis and immunoelectrophoresis were within normal limits. Test for anti-neutrophil cytoplasmic antibodies (ANCA) was positive for myeloperoxidase (MPO) and negative for proteinase 3 (PR3). Computed tomography (CT) demonstrated several bilateral peripheral nodular infiltrates in the lungs (Figure 1) and several nodular lesions in the liver and kidney; the spleen was mildly enlarged. Bone marrow biopsy was negative for granulomas and mycobacterium tuberculosis as well as for malignancy. The patient was discharged with a recommendation for ambulatory treatment with doxycycline for suspected atypical infection.

One month later, due to the ongoing fever, abdominal pain, repeated vomiting, constipation, weight loss, and loss of smelling ability, he was admitted again. Repeated CT scan showed resolution of previous pulmonary nodules with the concomitant appearance of several new ones. The number of hepatic nodules had increased with further enlargement of the spleen and the appearance of mild mesenteric lymphadenopathy. CT-guided fine needle aspiration of the pulmonary lesion failed and an open lung biopsy was performed. At that time, the patient was transferred to our hospital.

On admission the patient looked ill, pale, and weak. Heart and lung evaluation was unremarkable. There was mild tenderness of the upper abdomen without signs of peritoneal irritation or liver enlargement; the spleen was mildly enlarged and palpable. Repeated blood tests showed WBC 4200/*μ*L, HB 12.4 g/dL, PLT 248000/*μ*L, creatinine 0.65 mg/dL, albumin 3.9 g/dL, AST 25 U/L, ALT 43 U/L, GGT 52 U/L, ALKP 123 U/L, LDH 234 U/L, CK 20 U/L, CRP 16.43 mg/L, and ESR 24 mm/1 hour. Repeated ANA and anti-dsDNA were negative; the test for ANCA was pending. Gastroscopy showed only a small sliding hiatal hernia. Otolaryngologist assessment and brain magnetic resonance imaging were normal. Fundus evaluation did not show signs of retinal vasculitis. Transthoracic echocardiography was normal.

During the next several days the patient's condition deteriorated due to progressive weakness, abdominal pain, and repeated vomiting. After receiving the lung biopsy results which were summarized as necrotizing vasculitis and taking into account the existing findings from previous assessments (positive MPO, pulmonary nodules), the working diagnosis of ANCA-associated vasculitis was suggested and pulse therapy with methylprednisolone 1000 mg/day for 3 consecutive days was introduced. After the first infusion the patient continued to complain of abdominal pain, constipation, and vomiting, and he refused to eat. Serial clinical abdominal assessments revealed epigastric tenderness without signs of peritoneal irritation. X-ray did not show free air in the abdominal cavity or signs of bowel obstruction. Abdominal ultrasonography demonstrated multiple hypoechoic hepatic lesions with mild ascites. Suddenly, the patient developed acute and severe epigastric pain, diffuse abdominal wall rigidity and rebound, marked tachycardia, and hypotension.

**Figure 2:**
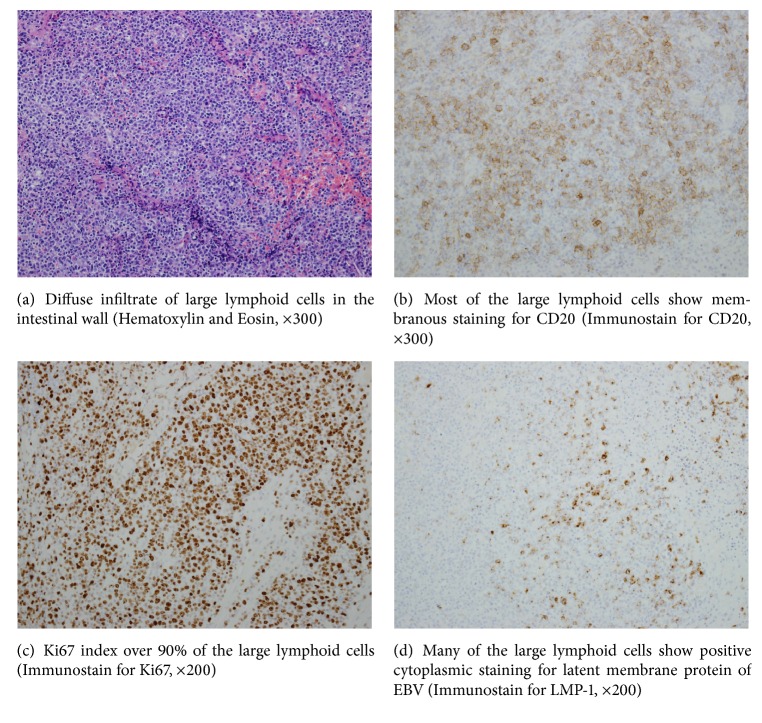
Histopathologic examination of intestinal biopsy.

CT angiography of the abdomen demonstrated large amount of free air in the upper abdomen, peritoneal effusion, and a thickened small and large bowel wall with no signs of mesenteric arteries or veins thrombosis. The patient underwent emergent laparotomy which revealed multiple small necrotic areas in different segments of the small bowel; the involved part of small bowel was resected. The postoperative period was unremarkable. Pathologic examination of the resected small bowel showed high-grade EBV-associated diffuse large B cell lymphoma with signs of lymphomatoid granulomatosis and positive IgH rearrangement (Figure 2). The intestinal wall and the blood vessels showed extensive lymphoid infiltration. Later, repeated ANCA revealed negative results for MPO and PR3. The patient was treated with the CHOP-R protocol (cyclophosphamide, doxorubicin, vincristine, prednisone, and rituximab) with achievement of rapid clinical and hematological remission.

## 3. Discussion

We describe a unique case of a young male who developed high-grade EBV-associated diffuse large B cell lymphoma mimicking granulomatosis with polyangiitis (GPA). Our patient presented with constitutional symptoms (prolonged fever, severe fatigue, and weight loss), nasal involvement (anosmia), multiple pulmonary, hepatic, and spleen nodules, elevated inflammatory markers, anemia, transiently positive MPO, and findings on open lung biopsy suggestive of necrotizing vasculitis. All together, these findings were “strong” clues for the diagnosis of ANCA-associated vasculitis.

GPA is a small and medium-sized vessels vasculitis which is highly associated with positive ANCA, mainly with antibodies against PR3 [1]. Among reported clinical manifestations of GPA, necrotizing granulomatous inflammation in the upper respiratory tract, pulmonary nodules (often with cavitations), and necrotizing glomerulonephritis are prominent; their combination is a classic GPA triad [1]. Involvement of other organs and systems, such as the skin, eyes, ears, and peripheral nervous system, is less specific but definitely is not rare. The involvement of gastrointestinal tract (GIT) in GPA is infrequent [2]. Akbulut reported a patient with severe GPA who developed bowel perforation and sepsis and eventually died. The author reviewed thirteen similar cases and summarized the main clinical features of GIT involvement in GPA as follows: gingivitis, peptic ulcers, bloody diarrhea, bowel ulcerations, persistent perianal ulcers, pancreatitis, cholecystitis, small bowel perforations, and colitis [2]. Involvement of the GIT in GPA has been usually reported during relapses, in uncontrolled diseases, and late in the disease course (2-3 years later or more). The most common GIT complications in vasculitis in general and in GPA particularly are oral ulcers, intestinal ulcerations, bleeding, and perforations. In this regard, treatments such as corticosteroids and non-steroidal inflammatory drugs have been suggested as triggering agents. The presence of active vasculitis-related changes at the sites of bowel perforations in about 61% of reported cases supports the idea that uncontrolled vasculitis in the intestinal wall was the main reason for perforation and not corticosteroids as was presumed previously [2]. Involvement of the GIT as a presenting symptom of GPA is extremely rare. We could find in the English language literature only 20 cases with GIT involvement as an early (less than one-year disease duration) or even initial GPA presentation (Table 1). Patients were mainly males (15/20) with the mean age of 41.7 years (our patient was much younger) [3–22]. According to case descriptions, 4 (20%) patients had appetite and weight loss, 9 (45%) patients had oral and/or tongue ulcers, 6 (30%) had diarrhea, 9 (45%) had blood in stool, melena, or bloody diarrhea, 2 (10%) had anal ulcers, and 1 had odynophagia (2%); 9 (45%) patients developed “acute abdomen” due to perforation, intra-abdominal bleeding, or obstruction. Our patient had abdominal symptoms for more than two months but never had oral ulcers, diarrhea, or GIT hemorrhage. Only in one reported patient was the GIT involvement an isolated GPA feature but was accompanied by fever [3] while, in the majority of cases, there were signs of multiorgan disease with almost an obligatory involvement of the upper airways (our patients had anosmia but not involvement of sinuses, eyes, or ears) and lungs (our patient had nodules without cavitations) and less often the kidneys (our patient did not have signs of nephritis).

Walton analyzed clinical and pathology features in 56 cases of GPA (Wegener's granulomatosis at that time). He found granulomatous lesions in the spleen (55.6%), kidney (66.7), and liver (16.7%) and fibrinoid necrosis in blood vessels in the spleen (77.8%), kidney (77.8%), liver (18.5%), intestine (24.1%), pancreas, and gallbladder (7.4% each) [23]. One patient had severe refractory anosmia which later was complicated by nasal septum destruction and deafness (our patient also had anosmia but not structural or mucosal damage in the oral or nasal cavity). Described liver and spleen nodules were mainly microscopic findings but not macroscopic lesions on imaging films. Although the classic splenic manifestations of GPA are splenic infarcts, hemorrhage and spleen necrosis may also occur [24–27]. In one GPA case, multiple small bowel ulcers and perforations were accompanied by spleen infarctions [9]. Walton and Leggat described a patient with anosmia, lung involvement, multiorgan failure, and spleen granulomas [15].

Involvement of the liver is extremely rare in the course of GPA. Among liver manifestations, nonspecific nontraumatic hepatic hematoma and incomplete septal cirrhosis were reported [28, 29]. Holl-Ulrich and Klass described a patient with necrotizing granulomatous changes in the liver, lungs, parotid glands, and skin in a GPA patient with fatal liver failure [30]. Our patient had multiple nodules in the liver, spleen, and kidneys. Liver biopsy in our patient demonstrated interface hepatitis without granulomas.

Before the 1990s, the main tools for assessment of GPA patients were X-rays with contrast and endoscopy. CT imaging became available later and was found to be very useful. It was obvious from analyzed cases that endoscopy had limited value in the diagnosis of vasculitis, as the main findings were mucosal ulcerations which are not specific for GPA (our patient did not have mucosal damage on endoscopy). In cases with active GIT bleeding on endoscopy, the diagnosis of vasculitis was more reasonable. Inability to perform the deep transmural biopsy during endoscopy also limited the diagnostic value of endoscopy; in the majority of reported cases GPA diagnosis was confirmed in resected bowel specimens or, unfortunately, in autopsies. In the 1980s, the main immunologic abnormality was positive rheumatoid factor (three cases); later, ANCA became a standard diagnostic tool and was reported positive in all but one case, c-ANCA or PR3 in particular (our patient had a transient MPO positive test).

The definitive diagnosis of GPA is based on tissue biopsy, mainly from upper respiratory tract or lung tissue. It is very clear from the Walton report that the presence of granulomas with neutrophils accumulation is a prominent and specific sign, while vascular wall fibrinoid necrosis is nonspecific and probably could only reflect the damage to the blood vessel wall by granuloma [23].

The diversity of GPA clinical manifestations that includes systemic signs (sort of “B-signs”) and multiple organ and tissue involvement placed GPA in the list of great “imitators”, as it shares various clinical features with other systemic diseases such as hematologic malignances and infections, making the definite diagnosis very challenging. Given the fact that our patient had a combination of listed features, positive ANCA, and findings on open lung biopsy suggestive of necrotizing vasculitis, the working diagnosis of GPA was justified, as was treatment with glucocorticoid pulse therapy. Our patient developed intestinal perforations. “Acute abdomen” with intestinal perforations has been described in GPA patients with GIT involvement [9, 16, 17, 22]. In cases with acute abdomen, laparotomy is mandatory. In our patient, biopsies from resected bowel segments showed signs of diffuse large B cell lymphoma cells. Retrospective revision of lung pathology confirmed that the damage to the blood vessels was mainly due to an accumulation of lymphocytes in the involved tissue but not neutrophil accumulation or granulomas.

Different types of lymphoma may mimic different types of vasculitis. Lymphoma may be associated with ANCA positivity for both PR3 and MPO without vasculitis [31]. In a series of 119 patients with non-Hodgkin's lymphoma and 60 patients with Hodgkin's lymphoma, ANCA positivity was found in 8 patients (6 with p-ANCA and 2 with c-ANCA) with Hodgkin's lymphoma. None had vasculitis or rheumatic manifestations [32]. We could not explain the inconsistent results of positive MPO; in our institution the test for ANCA was negative. Despite the rare association between lymphoma and vasculitis, vasculitis in lymphoma patients mainly presents with skin purpuric rash and leukocytoclastic vasculitis on biopsy; our patient did not have any rash.

In searching the literature, we were able to find three cases of diffuse large B cell lymphoma mimicking GPA [33–35]. All these patients, like our patient, were males, aged between 20 and 40 years of age, and had pulmonary involvement; all had negative ANCA. None of these patients, in contrary to our patient, had liver or spleen involvement; one patient had renal involvement. In the case reported by Cohen et al., the patient was diagnosed with GPA and initially “responded” well to steroids and cyclophosphamide; only because of severe relapse under the treatment, revision of the biopsy leads to the proper diagnosis of lymphoma.

Did we have some clues to doubt the diagnosis of vasculitis in a patient who presented with systemic signs, multiorgan clinical presentations, presence of pulmonary nodules, positive MPO, and signs of vascular damage on open lung biopsy? A retrospective analysis of the case could teach us to pay attention to tiny discrepancies in the patient's condition: the anosmia in the absence of upper respiratory tract involvement was probably a “red herring”; the presence of multiple small and not consistent pulmonary nodules without cavitation is not a typical GPA feature; the presence of nodular lesions on CT imaging (“macroscopic”) in the spleen, kidneys, and liver without signs of hemorrhage is not typical for GPA; severe dyspepsia (abdominal pain, vomiting, and constipation) without any mucosal damage (ulceration or bleeding) on upper endoscopy could not be explained by vasculitis, especially in the absence of diarrhea or intestinal/rectal bleeding. GPA is a disease with neutrophils activation and accumulation in organs and tissues; our patient did not have leukocytosis. Also, he had only mildly elevated ESR and CRP in contrast to very high levels in patients with GPA. It is unusual not to find c-ANCA (especially PR3) in patients with active GPA and multiorgan involvement as in our patient. Finally, the absence of granulomas in the involved lung tissue with only the pathological finding of vessel wall damage should raise the question regarding a diagnosis of vasculitis or a search for an alternative diagnosis.

We believe that in cases of atypical clinical course of systemic illness suggestive of vasculitis (GPA), the differential diagnosis should include lymphoma. Prompt efforts should be invested to confirm the diagnosis of vasculitis or to ensure that another condition, such as lymphoma, is not the cause of the disease. Our report pointed out the difficulties and obstacles on the way to making the correct diagnosis in patients with B cell lymphoma mimicking systemic GPA-like vasculitis.

## Figures and Tables

**Table 1:** Analysis of cases with gastrointestinal symptoms in the early course of granulomatosis and polyangiitis (one year or less).

Author, year of publication	Gender, age (years)	Presenting symptoms	Disease duration	APR elevation	Concomitant respiratory system/kidney/other involvement	CT/endoscopic findings	Biopsy findings	ANCA
Yoshikawa et al., 2014 [3]	Male, 30	Oral ulcer, bloody stool	1 month	ESR, CRP	Fever	CT: thickening of the transverse colon wall	Superficial inflammatory cell infiltration and edema noted in lamina propria mucosae	PR3

Sinnott et al., 2013 [4]	Male, 29	Bloody diarrhea	2 months	ESR, CRP	Lung/kidney	CT: inflammatory changes in caecum and ascending colon	No bowel biopsy	PR3

Dag et al., 2013 [5]	Male, 29	Bloody stool, rectal bleeding	6 months	CRP, ESR	Lung, kidney, joints, skin	CT: distal ileum perforations Colonoscopy: ulcers in ileum, cecum, colon	Active chronic inflammation and ulcer bases	PR3

Qian et al., 2010 [6]	Female, 79	Bloody diarrhea	Presenting symptom	Not reported	Pulmonary hemorrhage, acute renal failure	Colonoscopy: pancolitis with numerous ulcers of varying sizes	Mucosal ulcerations with foci of acute inflammation and hemorrhage Kidney biopsy: crescentic and necrotizing glomerulonephritis	PR3

Samim et al., 2010 [7]	Male, 35	GI bleeding	1-2 weeks	CRP	Sinusitis/kidney/arthritis/skin rash	CTA: bleeding from one mesenteric artery Endoscopy: mucosal ulcerations	Central ulcerative inflammation and occluded small arteries in intestinal wall	PR3

Marie et al., 2010 [8]	Male, 31	Epigastric pain, rectal bleeding	Before diagnosis of GPA	ESR, CRP	Arthritis, purpura	Colonoscopy: multiple rectal and sigmoidal ulcerations with spontaneous bleeding	Inflammatory cell infiltrates surrounding small vessels	PR3

Deniz et al., 2007 [9]	Male, 44	Acute abdomen	4 weeks	Not reported	Sinusitis, lung cavitation nodules	CT: intra-abdominal free air and fluid, small bowel wall thickening, chronic splenic infarction	Ulcerations, necrotizing transmural granulomatous inflammation, fibrinoid necrosis of small to medium-sized submucosa vessels	ANCA

Kuwahara et al., 2006 [10]	Male, 30	Bloody diarrhea, oral and anal ulcers	Before diagnosis of GPA	CRP	Nasal bleeding, skin and mucosal ulcers, conjunctivitis	Colonoscopy: multiple ulcerations with irregular shapes, macroscopic bleeding, pseudopolyposis in terminal ileum, colon, rectum	Inflammation and epithelioid granuloma with multinuclear giant cells	PR3

Socas Macías et al., 2005 [11]	Male, 28	Oral ulcers, diarrhea, recurrent bowel obstruction	Before diagnosis of GPA	Not reported	Nasal and upper airway, skin rash with necrosis, glomerulonephritis	Colonoscopy and GIT X-rays: ulcers and structures due to ulceration in terminal ileum and colon (signs compatible with Crohn's disease)	Endoscopic biopsy: features compatible with Crohn's disease in large bowel and terminal ileum Autopsy: small vessel vasculitis (arterioles and venules), occasional histiocytes around vessels forming loose granulomas with scattered multinuclear giant cells	Negative

Chow et al., 2003 [12]	Male, 46	Melena	4 weeks	Not reported	Sinusitis, glomerulonephritis	Endoscopy: patchy ulceration of small bowel mucosa Nuclear medicine scan: proximal jejunal blood loss	Resected jejunum: small vessel vasculitis of bowel, mucosal ulceration, submucosa inflammation, fibrosis	c-ANCA

Fallows et al., 2000 [13]	Female, 34	Odynophagia	4 weeks	ESR	Otitis, conjunctivitis, skin rash and ischemia, arthritis, mild kidney involvement	Esophagoscopy: multiple punch-out ulcers	Fibrinoid necrosis with ulcerations and inflammatory cells infiltration surrounding small blood vessels	c-ANCA

Shaikh et al., 2006 [14]	Male, 44	Abdominal pain, peritonitis	Several weeks	ESR	Polyarthralgia, skin rash, polyneuropathy	X-ray and abdominal CT: pneumoperitoneum Laparotomy (three times): multiple perforations in ileum, colonic ischemia	Resected ileum: vasculitis with fibrinoid necrosis	c-ANCA

Walton and Leggat, 1956 [15]	Female, 42	Oral and tongue ulcers	10 months	Not reported	Fever, anosmia and nasal ulcers, deafness, lung infiltrates and pleurisy, arthritis, rash, neuritis, kidney involvement	ND	Autopsy (data restricted to GIT): multiple spleen solitary granulomata similar to those in lung, signs of vasculitis in pancreas	ND

Tokuda et al., 1989 [16]	Male, 37	Gingival ulcers, weight loss, abdominal pain, distention, ascites, peritonitis due to ileum perforation	Nasal symptoms 2 years; systemic disease several weeks	CRP	Rhinorrhea, arthralgia, fever, nasal blockade, necrotizing glomerulonephritis	X-ray: abdominal ascites	Biopsy from perforated ileum, granulomatous vasculitis	ND

Srinivasan and Coughlan, 1999 [17]	Female, 56	Crampy abdominal pain, perforation, weight loss	8 weeks	ESR, CRP	Rhinorrhea, arthralgia, fever, nasal bridge swelling	Barium study: normal Laparotomy: areas of perforation	Granulomatous reaction surrounding perforation without inflammation	c-ANCA

Geraghty et al., 1986 [18]	Male, 46	Weight loss, palate ulcerations, peritonitis	4–8 weeks	ESR	Fever, lung and kidney involvement, fingers and toes ischemia	Chest X-ray: free air in abdominal cavity Laparotomy: inflammation of entire small bowel with numerous areas of necrotic ulceration, multiple perforations in terminal ileum	Autopsy: large perforations of ascending colon and multiple punched out ulcers of distal small bowel and proximal large bowel; granulomata in lungs, spleen, and prostate	RF

Haworth and Pusey, 1984 [19]	Female, 43	Mouth ulcer, bloody mucous diarrhea, weight loss, anorexia	11 months	Not reported	Sinusitis, otitis, deafness, arthritis, iritis, rash, kidney involvement	Sigmoidoscopy: ulcerated rectal mucosa with spontaneous bleeding Barium meal: narrowed terminal ileum and cecum Barium enema: ulcerated and ragged cecum	Biopsy: neutrophilic infiltration in rectal and sigmoid mucosa	RF

McNabb et al., 1982 [20]	Male, 50	Pharyngeal and mouth ulcers, abdominal pain, vomiting, peritonitis	Nine months symptoms, GIT symptoms a week after diagnosis	ESR	Arthralgia, nasal blockade, epistaxis, pleurisy, hemoptysis, fingers nodules, proteinuria, renal failure	Laparotomy: multiple ileum ulcers	Biopsy: nonspecific ulcerations	RF

Aymard et al., 1990 [21]	Male, 46	Anal ulcerations	Six months before systemic vasculitis	ESR	Weight loss, polyarthralgia, otalgia, hearing loss, lt. peripheral fascial nerve palsy, hemoptysis	Colonoscopy: normal X-rays of small intestine normal Chest X-ray: right pulmonary apical lesion with cavitation CT: mastoiditis and otitis media	Necrotizing vasculitis, ulcerations and microgranuloma	ANCA

Akça et al., 2005 [22]	Male, 56	Tongue plaques, intestinal perforation	Before diagnosis	ESR, CRP	Pulmonary nodules, severe skin vasculitis	Chest and abdomen X-ray: free air under the diaphragm	Mucosal ulceration, mixed inflammatory cells infiltration and transmural infarcts	c-ANCA

*Notes*. APR = acute phase reactant; CT = computed tomography; ANCA = anti-neutrophil cytoplasmic antibody; ESR = erythrocyte sedimentation rate; CRP = C-reactive protein; PR3 = proteinase 3; GI = gastrointestinal; CTA = computed tomographic angiography; GPA = granulomatosis with polyangiitis; c-ANCA = cytoplasmic anti-neutrophil cytoplasmic antibody; ND = not done.
